# Alterations in Tryptophan Metabolism and the Indole Pathway in Colorectal Cancer Patients: A Systematic Review and Meta‐analysis

**DOI:** 10.1002/mco2.70466

**Published:** 2025-11-20

**Authors:** Liam Griffith, Akshat Sinha, Animesh Acharjee

**Affiliations:** ^1^ Department of Cancer and Genomic Sciences, School of Medical Sciences, College of Medicine and Health University of Birmingham Birmingham UK; ^2^ MRC Health Data Research UK (HDR UK) Birmingham UK; ^3^ Institute of Translational Medicine University Hospitals Birmingham NHS Foundation Trust Birmingham UK; ^4^ Centre For Health Data Research University of Birmingham Birmingham UK

1

Dear Editor,

Colorectal cancer (CRC) poses a significant threat to global health, with early detection and treatment dramatically improving survival rates [1]. CRC arises from a combination of both hereditary and environmental factors, with the gut microbiome being increasingly recognised as a key driver of disease. Tryptophan metabolism is implicated in CRC development through the serotonin, kynurenine, and indole pathways. The indole pathway, which is mediated by the gut microbiome, has showcased anti‐tumour and anti‐inflammatory properties in vitro, but remains relatively under‐researched in humans. To explore alterations in tryptophan metabolism and the indole pathway in CRC, we conducted a systematic review and meta‐analysis according to the Preferred Reporting Items for Systematic Reviews and Meta‐Analyses (PRISMA) statement and Cochrane Handbook for Systematic Reviews of Interventions version 6.4 [2]. This review was registered prospectively with the International Prospective Register of Systematic Reviews (PROSPERO), registration ID: CRD42024509207.

Ovid Medline, Ovid Embase, PubMed, Scopus, and Web of Science were searched from inception up to January 5 2024, including both forward and backwards searching of citation networks. Tryptophan and indole derivative fold change values between colorectal cancer patients and suitable controls were extracted from observational studies only. Samples analysed included plasma, faecal, colonic tissue, and urine; these samples were analysed independently using the same statistical framework and were not pooled across biofluids. Only studies published within the last 15 years were included, and grounds of exclusion included comorbidities or diseases other than CRC, studies that lacked sufficient tryptophan or indole analysis, studies lacking relevance to the research question, animal or in vitro studies, and studies without an available full text (Table S1). Risk of bias was assessed using ROBINS‐E [3], and publication bias through funnel plots and Egger's test. Meta‐analyses employed random‐effects models to synthesise fold‐change values. Pathway impact scores were analysed narratively to assess the physiological impact of metabolic pathway alterations, by factoring in affected metabolites, as well as their relative importance in the pathway. Indole derivative fold change values were unsuitable for meta‐analysis and were therefore reviewed narratively. Biomarker utility was evaluated narratively using area under the curve (AUC) metrics.

Of 1672 studies screened, 21 met inclusion criteria, including 23 cohorts (21 case‐control, one nested case‐control, and one prospective cohort) containing 6719 participants (Table S2, S3). Plasma Tryptophan levels significantly decreased in CRC patients (log2 Fold Change [log2FC] = ‐0.3203; 95% Confidence Interval [CI] [‐0.5737; ‐0.0668], *p* = 0.0133) (Figure 1). Subgroup analysis suggests this decrease may only be present in Asian cohorts and not European; more data is needed to confirm this, as the analysis lacked statistical power.

**FIGURE 1:**
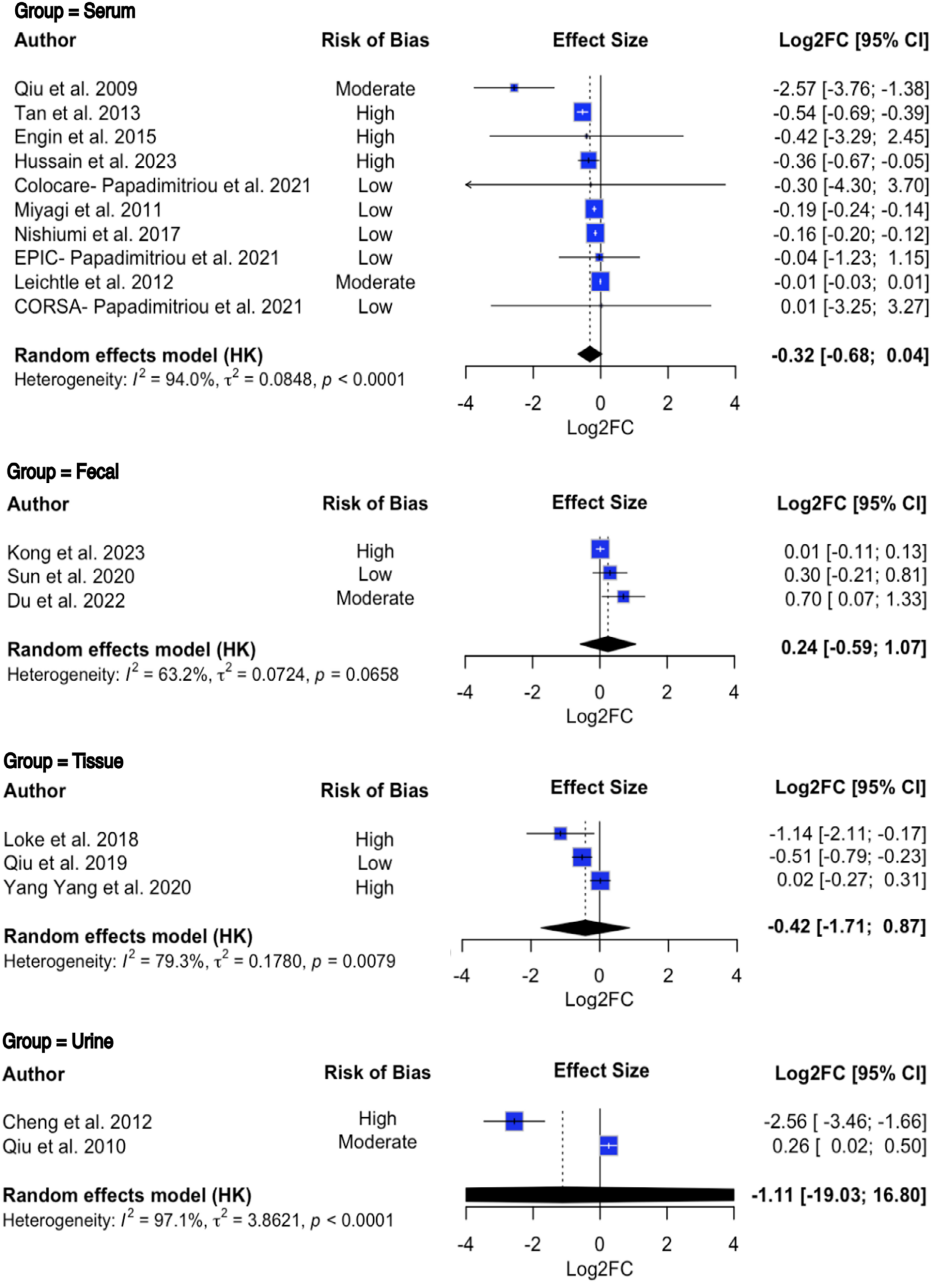
Forest plot depicting the log‐transformed fold change in plasma, faecal, tissue and urine tryptophan levels in colorectal cancer patients compared to controls. Error bars represent the 95% confidence interval around the true effect size. Each square denotes the effect size from the corresponding study, with the size of the squares corresponding to the relative weight of each study. The black diamond represents the overall effect size, accounting for all the studies included in the analysis. The black bar beneath the diamond denotes the prediction interval representing the 95% confidence interval of the results. Random‐effect meta‐analysis was conducted using the restricted maximum likelihood model. Each study had its respective weight calculated based on the precision of its effect size; this determined what percentage of the overall pooled result a given study would contribute to. No pooling or direct comparisons of Log2FC values across biofluids were performed, as we acknowledge that such comparisons would assume cross‐biofluid equivalence, which is methodologically inappropriate without specific normalisation. The biofluids are presented together in Figure 1 for interpretability only. Log2FC = log‐transformed fold change, 95% CI = 95% confidence interval.

Faecal, Tissue and Urine Tryptophan samples showed no significant changes (log2FC = 0.2410; 95% CI = [‐0.1407; 0.6228], *p* = 0.2159, log2FC = ‐0.4142; 95% CI = [‐0.9714; 0.1430], *p* = 0.1451, and log2FC = ‐1.1158; 95% CI = [‐3.8863; 1.6546], *p* = 0.4299, respectively) (Figure 1). Both Higgins and Thompson's I2 statistic and Cochran's Q test found the Tissue and Urinary data to have very high between‐study heterogeneity.

Our findings of a significant decrease in plasma Tryptophan align with previous reviews on the topic [4]. Several in vitro studies have reported tryptophan depletion in CRC due to the overexpression of IDO1, and subsequent overactivity of the kynurenine pathway. Tryptophan depletion suppresses T and natural killer cell function, promotes Treg proliferation, and drives tumour angiogenesis; the physiological importance of tryptophan depletion is further highlighted by human trials reporting that reduced plasma tryptophan correlates with diminished quality of life and prognosis in CRC patients [5].

Eight indole metabolites exhibited significant alterations. Most notably, all studies found IA, IAA, and IPA to be downregulated in CRC. This has numerous anti‐tumour implications, such as decreased AHR activity, proinflammatory cytokine signalling, loss of epithelial integrity, a shift in immune populations toward pro‐inflammatory phenotypes, and increased leukocyte trafficking. Alternatively, IAld and I3CA were upregulated; this has been shown to downregulate NF‐KB signalling and upregulate IL‐22 secretion and STAT3 signalling activity, all of which elicit anti‐tumour effects. This contradiction may be explained by the ability of AHR agonists to drive the proliferation of Treg cells, and therefore the promotion of immune evasion. These findings contradict our understanding of their role in tumorigenesis and require further exploration. Overall, current literature suggests there are significant alterations in indole derivative production in CRC; further study of these compounds in CRC is highly recommended.

Three of the included studies also reported pathway impact scores, which reflect both the number of altered metabolites within a pathway and their biological importance. Two of these studies identified significant disruptions in tryptophan metabolism. One reported a moderate impact score of 0.17 (95% CI = [‐0.01; 0.35], *p* = 0.03), indicating a borderline but statistically significant perturbation. The other found a slightly lower impact score of 0.11 (0.11; 95% CI = [0.03; 0.18], *p* = 0.0045), but with stronger statistical confidence, suggesting a reliable disruption of this metabolic pathway. Five studies assessed the validity of Tryptophan as a biomarker in the diagnosis of CRC, as well as its performance as part of a multi‐variate diagnostic panel. Our results found Tryptophan to perform moderately well as a standalone biomarker. As part of a multi‐parameter diagnostic panel, Tryptophan may show diagnostic potential, with one study's multi‐parameter panel returning an AUC of 0.996.

This study had several strengths and limitations. The inclusion of plasma results from 10 studies and 4098 participants provided robust insight into alterations in plasma Tryptophan levels among CRC patients. The comprehensive systematic search conducted during this review spanned several databases, reducing the likelihood of missing eligible data. Finally, this review combined several insights into tryptophan metabolism to create an easily interpretable synthesis. However, a major limitation in conducting this review was the impact of small cohorts. Several analyses in this study were conducted with small sample sizes, introducing large amounts of heterogeneity and reducing statistical power; this was particularly apparent in the faecal, tissue, and urinary meta‐analyses. Furthermore, while many of the included studies obtained samples prior to the commencement of treatment, many did not specify if this was the case; chemotherapy or alternative treatments may have impacted the gut microbiome or tryptophan metabolism, therefore impacting our findings.

Regarding future directions, multi‐variate diagnostics containing Tryptophan show potential, and should be investigated further prior to clinical testing; in order to optimally identify disease stage and prognosis, researchers should aim to integrate these markers into multi‐omic panels; combining genotypic and phenotypic data can enable successful tumour subtyping, prognostic analysis, and optimal treatment. Our study also identified the need for further investigation into indole derivatives; despite the growing body of evidence supporting their anti‐tumour effects and clinical potential, these compounds remain under‐researched. Future studies investigating indole derivatives should include both absolute concentrations and AUC values where possible, in order to evaluate their diagnostic potential. Furthermore, since each included study only analysed one sample type, future studies may consider including multi‐tissue analyses to provide further insight. More data on faecal, tissue and urinary Tryptophan is needed, and Tryptophan metabolism in Asian cohorts specifically should be reviewed further.

In conclusion, fold‐change and pathway impact analysis found tryptophan metabolism to be significantly altered in CRC. Furthermore, several key indole derivatives with anti‐tumour properties are downregulated. However, several tumour‐promoting indole derivatives were unexpectedly upregulated. Biomarker analysis found Tryptophan‐containing multi‐variate panels to show potential in the diagnosis of CRC. Tryptophan and the indole pathway may show promise regarding potential therapeutics and diagnostics in CRC, although data are sparse.

## Author Contributions

A.A. conceptualised, supervised and designed the project. L.G. contributed to the analysis of the public data and, literature review. A.S. provided clinical and biological interpretation and literature review. L.G. and A.A. wrote the first draft. All authors have accepted responsibility for the entire content of this manuscript and approved its submission.

## Funding

The authors acknowledge support from the MRC Health Data Research UK (HDRUK/CFC/01), an initiative funded by UK Research and Innovation, Department of Health and Social Care (England), and the devolved administrations, and leading medical research charities. The views expressed in this publication are those of the authors and not necessarily those of the NHS, the National Institute for Health Research, the Medical Research Council, or the Department of Health.

### Ethics Statement

No ethical approval was required for this study.

## Conflicts of Interest

The authors declare that they have no known competing financial interests or personal relationships that could have appeared to influence the work reported in this paper.

## Supporting information




**TABLE S1**: Studies excluded after full text review and their reason for exclusion, following the search criteria outlined in the studies' PROSPERO registration.
**TABLE S2**: Study characteristics table. CRC = Colorectal cancer patients, HC = Healthy control, LC‐MS = Liquid Chromatography Mass Spectrometry, BMI = Body Mass Index, Log2FC = Log2 Fold Change.
**TABLE S3**: Protocol Deviations from PROSPERO Registration. We acknowledge some deviations from our registered PROSPERO protocol (CRD42024509207), which are outlined in this table. All changes were made prior to data extraction or analysis and were not based on knowledge of study outcomes.

## Data Availability

All data sets are publicly available.
