# Supplementary material for: Alterations in Tryptophan Metabolism and the Indole Pathway in Colorectal Cancer Patients: A Systematic Review and Meta‐analysis
Source: MedComm (2020). 2025 Nov 20;6(12):e70466. doi: 10.1002/mco2.70466 (PMC12635423; doi:10.1002/mco2.70466)
Supplement: Supplementary file 1 — TABLE S1: Studies excluded after full text review and their reason for exclusion, following the search criteria outlined in the studies' PROSPERO registration. TABLE S2: Study characteristics table. CRC = Colorectal cancer patients, HC = Healthy control, LC‐MS = Liquid Chromatography Mass Spectrometry, BMI = Body Mass Index, Log2FC = Log2 Fold Change. TABLE S3: Protocol Deviations from PROSPERO Registration. We acknowledge some deviations from our registered PROSPERO protocol (CRD42024509207), which are outlined in this table. All changes were made prior to data extraction or analysis and were not based on knowledge of study outcomes. [file MCO2-6-e70466-s001.docx]

**Supplementary materials**

**Alterations in tryptophan metabolism and the indole pathway in colorectal cancer patients: A systematic review and meta-analysis**

**Liam Griffith ^1^, Akshat Sinha ^1^, Animesh Acharjee ^1,2,3,4*^**

^1^ Cancer and Genomic Sciences, School of Medical Sciences, College of Medicine and Health, University of Birmingham, Birmingham, UK

^2^ MRC Health Data Research UK (HDR UK), Birmingham, UK

^3^ Institute of Translational Medicine, University Hospitals Birmingham NHS, Foundation Trust, UK

^4^ Centre for Health Data Research, University of Birmingham, United Kingdom

***Correspondence**

Dr. Animesh Acharjee

Associate Professor in Integrative Analytics and AI

Institute of Cancer & Genomic Sciences

University of Birmingham, B15 2TT, UK

**E-mail:** [a.acharjee@bham.ac.uk](mailto:a.acharjee@bham.ac.uk)

**Phone:** +44 121 414 7012

Supplementary table 1. Studies excluded after full text review and their reason for exclusion, following the search criteria outlined in the studies PROSPERO registration.

| \| Author. Date \| Study title \| Reason for exclusion \| \| --- \| --- \| --- \| \| Baxter et al. 2019 \| Navy beans impact the stool metabolome and metabolic pathways for colon health in cancer survivors \| Study lacked tryptophan analysis \| \| Ewa et al. 2023 \| Global proteomic identifies multiple cancer-related signalling pathways altered by a gut pathobiont associated with colorectal cancer \| Study lacked tryptophan analysis \| \| Geraghty et al. 2023 \| Aspirin Produces Unique Plasma Metabolomic Signatures And Alterations Of Gut Microbial Tryptophan Metabolism: A Randomized Clinical Trial \| Study lacked tryptophan analysis \| \| Goedert et al. 2014. \| Fecal metabolomics: assay performance and association with colorectal cancer \| Study lacked tryptophan analysis \| \| Gonzalez-Mercado. 2021 \| Co-occurence of Symptoms and Gut Microbiota Composition Before Neoadjuvant Chemotherapy and Radiation Therapy for Rectal Cancer: A Proof of Concept \| Study lacked tryptophan analysis \| \| Han et al. 2023 \| Microbiota-derived tryptophan catabolites mediate the chemopreventitive effects of statins on colorectal cancer \| Study lacked tryptophan analysis \| \| Jassim et al. 2022 \| Escherichia coli Biomarker Types in Colorectal Cancer Patients \| Study lacked tryptophan analysis \| \| Jin et al. 2020 \| Prediction of Postoperative Ileus in Patients With Colorectal Cancer by Preoperative Gut Microbiota. \| Study lacked tryptophan analysis \| \| Kayano et al. 2019 \| Short- And Long-term Outcomes of 2-Step Stapled Intracorporeal Versus Extracorporeal Anastomosis in Laparoscopic Colectomy for Colon Cancer. \| Study lacked tryptophan analysis \| \| Laghi et al. 2018 \| Impact of Treatments on Fecal Microbiota and Fecal Metabolome in Symptomatic Uncomplicated Diverticular Disease of the Colon: A Pilot Study. \| Study lacked tryptophan analysis \| \| Marcial et al. 2017 \| Modulates the Host Immune Responses: A Double-Blind, Randomized Trial in Healthy Adults \| Study lacked tryptophan analysis \| \| Mohseni et al. 2023 \| Gut Microbiota and Its Metabolites Are Important Determinants of the Immune Response to the MUC1 Vaccine in the Setting of Colon Cancer Prevention \| Study lacked tryptophan analysis \| \| Mutignani et al. 2021 \| Blood Bacterial DNA Load and Profiling Differ in Colorectal Cancer Patients Compared to Tumor-Free Controls. \| Study lacked tryptophan analysis \| \| Nordin et al. 2023 \| Effects of FODMAPs and Gluten on Gut Microbiota and Their Association with the Metabolome in Irritable Bowel Syndrome: A Double-Blind, Randomized, Cross-Over Intervention Study. \| Study lacked tryptophan analysis \| \| Obermoser et al. 2023 \| Positive Effects of Probiotic Therapy in Patients with Post-Infectious Fatigue. \| Study lacked tryptophan analysis \| \| Ohara et al. 2010 \| Possibility of preventing colorectal carcinogenesis with probiotics. \| Study lacked tryptophan analysis \| \| Presti et al. 2021 \| A Randomized, Placebo-Controlled Trial Assessing the Effect of VISBIOME ES Probiotic in People with HIV on Antiretroviral Therapy \| Study lacked tryptophan analysis \| \| Ramos et al. 2019 \| Effect of prebiotic (fructooligosaccharide) on uremic toxins of chronic kidney disease patients: a randomized controlled trial \| Study lacked tryptophan analysis \| \| Shi et al. 2023 \| Altered Intestinal Microbial Flora and Metabolism in Patients with Idiopathic Membranous Nephropathy. \| Study lacked tryptophan analysis \| \| Sinha et al. 2016 \| Fecal Microbiota, Fecal Metabolome, and Colorectal Cancer Interrelations \| Study lacked tryptophan analysis \| \| Speciani et al. 2023 \| Garlic consumption in relation to colorectal cancer risk and to alterations of blood bacterial DNA \| Study lacked tryptophan analysis \| \| Wang et al. 2014 \| Label-free detection of serum proteins using surface-enhanced Raman spectroscopy for colorectal cancer screening \| Study lacked tryptophan analysis \| \| Wang et al. 2023 \| Constipation is associated with depression of any severity, but not with suicidal ideation: insights from a large cross-sectional study. \| Study lacked tryptophan analysis \| \| Xie et al. 2023 \| Metabolomic analysis of gut metabolites in patients with colorectal cancer: Association with disease development and outcome. \| Study lacked tryptophan analysis \| \| Yamamoto et al. 2019 \| Metabolomics reveals elevated urinary excretion of collagen degradation and epithelial cell turnover products in irritable bowel syndrome patients \| Study lacked tryptophan analysis \| \| Yarur et al. 2023 \| Higher Intra-Abdominal Visceral Adipose Tissue Mass Is Associated With Lower Rates of Clinical and Endoscopic Remission in Patients With Inflammatory Bowel Diseases Initiating Biologic Therapy: Results of the Constellation Study. \| Study lacked tryptophan analysis \| \| Zhang et al. 2023 \| Modulating a prebiotic food source influences inflammation and immune-regulating gut microbes and metabolites: insights from the BE GONE trial \| Study lacked tryptophan analysis \| \| Neis et al. 2017 \| Human splanchnic amino-acid metabolism \| Disease other than CRC \| \| Nikolaus et al. 2017 \| Increased Tryptophan Metabolism Is Associated With Activity of Inflammatory Bowel Diseases \| Disease other than CRC \| \| Plantinga et al. 2023 \| Exploration of associations among dietary tryptophan, microbiome composition and function, and symptom severity in irritable bowel syndrome. \| Disease other than CRC \| \| Sarnoff et al. 2023 \| A multi-omic brain gut microbiome signature differs between IBS subjects with different bowel habits \| Disease other than CRC \| \| Sheflin et al. 2015 \| Pilot Dietary Intervention with Heat-Stabilized Rice Bran Modulates Stool Microbiota and Metabolites in Healthy Adults \| Disease other than CRC \| \| Tian et al. 2014 \| Dynamic analysis of the endogenous metabolites in depressed patients treated with TCM formula Xiaoyaosan using urinary H NMR-based metabolomics. \| Disease other than CRC \| \| Yokoyama et al. 2022 \| Impaired tryptophan metabolism in the gastrointestinal tract of patients with critical coronavirus disease 2019 \| Disease other than CRC \| \| Alles et al. 1999 \| Effect of transgalactooligosaccharides on the composition of the human intestinal microflora and on putative risk markers for colon cancer \| Study more than 15 years old \| \| Chung et al. 1975 \| Tryptophanase of fecal flora as a possible factor in the etiology of colon cancer. \| Study more than 15 years old \| \| Karlin et al. 1985 \| Fecal skatole and indole and breath methane and hydrogen in patients with large bowel polyps or cancer. \| Study more than 15 years old \| \| Zuccato et al. 1993 \| Role of bile acids and metabolic activity of colonic bacteria in increased risk of colon cancer after cholecystectomy. \| Study more than 15 years old \| |
| --- | --- | --- | --- | --- | --- | --- | --- | --- | --- | --- | --- | --- | --- | --- | --- | --- | --- | --- | --- | --- | --- | --- | --- | --- | --- | --- | --- | --- | --- | --- | --- | --- | --- | --- | --- | --- | --- | --- | --- | --- | --- | --- | --- | --- | --- | --- | --- | --- | --- | --- | --- | --- | --- | --- | --- | --- | --- | --- | --- | --- | --- | --- | --- | --- | --- | --- | --- | --- | --- | --- | --- | --- | --- | --- | --- | --- | --- | --- | --- | --- | --- | --- | --- | --- | --- | --- | --- | --- | --- | --- | --- | --- | --- | --- | --- | --- | --- | --- | --- | --- | --- | --- | --- | --- | --- | --- | --- | --- | --- | --- | --- | --- | --- | --- | --- | --- | --- |

Supplementary table 2. Study characteristics table. CRC= Colorectal cancer patients, HC= Healthy control, LC-MS= Liquid Chromatography Mass Spectrometry, BMI= Body Mass Index, Log2FC= Log2 Fold Change.

| \|  \|  \|  \|  \|  \| \| --- \| --- \| --- \| --- \| --- \|   Study | Sample | Study location | Sample size | Mean age | Assay used | Control definition | Uncontrolled covariates | Log2FC | P Value |
| --- | --- | --- | --- | --- | --- | --- | --- | --- | --- | --- | --- | --- | --- | --- |
| Hussain et al. 2023 | Serum | China | n= 233 (94 CRC, 139 HC) | 57.3 CRC 57.3 HC | LC-MS | Healthy matched control | BMI, Diet, Smoking | -0.36 | <0.001 |
| Colocare- Papadimitriou et al. 2021 | Serum | Germany | n= 254 (153 CRC, 101 HC) | 65 CRC 51 HC | LC-MS | Healthy matched control | BMI, Diet | -0.3 | 0.007 |
| CORSA- Papadimitriou et al. 2021 | Serum | Austria | n= 436 (390 CRC, 46 HC) | 69 CRC 63 HC | LC-MS | Healthy matched control | BMI, Diet | 0.01 | 0.197 |
| EPIC- Papadimitriou et al. 2021 | Serum | Europe | n= 912 (456 CRC, 456 HC) | 56 CRC 56 HC | LC-MS | Healthy matched control | BMI, Diet | -0.04 | 0.042 |
| Nishiumi et al. 2017 | Serum | Japan | n= 573 (291 CRC, 282 HC) | 67 CRC 68 HC | LC-MS | Healthy matched control | Diet | -0.16 | <0.001 |
| Engin et al. 2015 | Serum | Turkey | n= 205 (97 CRC, 108 HC) | 61.7 CRC 55.5 HC | LC-MS | Healthy matched control | Age, Diet, Gender, Smoking | -0.42 | <0.001 |
| Tan et al. 2013 | Serum | China | n=203 (101 CRC, 102 HC) | 60.75 CRC 58.03 HC | LC-MS | Healthy matched control | BMI, Diet, Smoking | -0.54 | <0.001 |
| Miyagi et al. 2011 | Serum | Japan | n= 1194 (199 CRC, 995 HC) | 63.7 CRC 62.4 HC | LC-MS | Healthy matched control | N/A | -0.19 | <0.001 |
| Qiu et al. 2009 | Serum | China | n=129 (64 CRC, 65 HC) | 59 CRC 55 HC | LC-MS | Healthy matched control | Diet, Smoking | -2.57 | <0.001 |
| Kong et al. 2023 | Fecal | China | n= 441 (244 CRC, 197 HC) | 63.16 LO 60.91 LO-HC 40.88 EO 36.92 EO-HC | LC-MS | Healthy matched control | BMI, Diet, Smoking | 0.01 | <0.001 |
| Du et al. 2022 | Fecal | China | n = 66 (30 CRC 36 HC) | 56.67 CRC 59.27 HC | LC-MS | Healthy matched control | Diet | 0.7 | 0.03 |
| Sun et al. 2020 | Fecal | China | n= 67 (39 CRC, 28 HC | 63.63 CRC 56.85 HC | LC-MS | Healthy matched control | Diet, Smoking | 0.3 | 0.118 |
| Yang et al. 2020 | Tissue | China | n= 94 | 60 | LC-MS | Adjacent non-cancerous tissue | BMI, Diet, Smoking | 0.02 | >0.05 |
| Wang et al. 2019 | Tissue | China | n= 34 | 59.2 | LC-MS | Adjacent non-cancerous tissue | BMI, Diet | -0.51 | <0.001 |
| Loke et al. 2018 | Tissue | Malaysia | n= 17 | 62.47 | LC-MS | Adjacent non-cancerous tissue | BMI, Diet, Smoking | -1.14 | 0.022 |
| Cheng et al 2012 | Urine | China | n= 204 (101 CRC, 103 HC) | 59 CRC 60 HC | LC-MS | Healthy matched control | BMI, Diet, Smoking | -2.56 | <0.001 |
| Qiu et al. 2010 | Urine | China | n= 123 (60 CRC, 63 HC) | 58.8 CRC 55.5 HC | LC-MS | Healthy matched control | Diet, Smoking | 0.26 | 0.031 |

Supplementary table 3. Protocol Deviations from PROSPERO Registration. We acknowledge some deviations from our registered PROSPERO protocol (CRD42024509207) which are outlined in this table. All changes were made prior to data extraction or analysis and were not based on knowledge of study outcomes.

| Protocol item | Registered PROSPERO plan | Final practice | Justification |
| --- | --- | --- | --- |
| Study publication date limits | Include studies up to 10 years old | Included studies up to 15 years old | Extended to increase the number of eligible studies and capture more relevant data |
| Study design inclusion | Include both randomised controlled trials (RCTs) and observational studies | Included only observational studies | No RCTs were found and observational studies were found to more accurate answer the research question |
| Risk of Bias tools | RoB2 | ROBINS-E | Changed to reflect study type used |
| Effect size metric | Mean difference | Log2FC | Log2FC was already reported in studies and is standard practice in metabolomic analyses |
